# 189. Outcomes of High-dose Oral Beta-lactam Step-down Therapy Compared to Alternative Oral Therapy for Bacteremic Urinary Tract Infections

**DOI:** 10.1093/ofid/ofad500.262

**Published:** 2023-11-27

**Authors:** Abigail Geyer, Lisa E Dumkow, Kali VanLangen, Andrew Jameson

**Affiliations:** Trinity Health Grand Rapids, Grand Rapids, Michigan; Trinity Health Grand Rapids, Grand Rapids, Michigan; Ferris State University College of Pharmacy, Grand Rapids, Michigan; Trinity Health Grand Rapids, Grand Rapids, Michigan

## Abstract

**Background:**

Pyelonephritis and associated bacteremia are often treated with intravenous antibiotics followed by oral step-down therapy to a fluoroquinolone (FQ) or trimethoprim/sulfamethoxazole (TMP/SMX). Oral beta-lactams were historically utilized less often due to concern for treatment failure. Literature suggests oral beta-lactams are effective, but there are no preferred oral beta-lactam agents or dosing recommendations. This study evaluates outcomes of high-dose oral beta-lactam therapy for Enterobacterales bacteremia due to a urinary tract infection (UTI) compared to alternative therapy.

**Methods:**

A retrospective, multicenter, observational cohort study was conducted evaluating patients admitted from February 1, 2020, to October 1, 2022, with gram-negative bacteremia from a urinary source. Adult patients were included if they received empiric intravenous antibiotic active against the bacteria isolated and transitioned to appropriately dosed oral cephalexin, amoxicillin, TMP/SMX, or FQ (Table 1). Excluded were patients who received less than 72 hours of oral therapy, had a diagnosis of renal abscess or lobar nephronia, and those who expired during admission. The primary outcome compared the composite of recurrent bacteremia or mortality within 30 days of completing oral therapy. Secondary outcomes included recurrent UTI, emergency department (ED) or hospital readmission, and *Clostridioides difficile* infections within 30 days of treatment completion.

**Figure:**
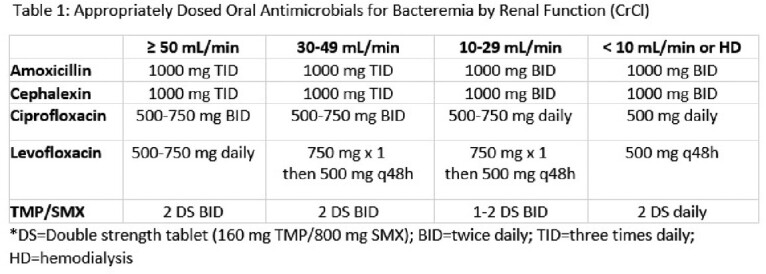

**Results:**

There were 194 patients who met inclusion criteria. The primary outcome occurred in 1/75 (1.3%) patients in the oral beta-lactam group and 2/119 (1.7%) patients in the FQ or TMP/SMX group (p=1.0). Two patients in the FQ or TMP/SMX expired within 30 days and 1 patient in the beta-lactam group had a recurrent bacteremia. There were 16 (21.3%) and 14 (11.8%) patients in the beta-lactam and FQ or TMP/SMX group, respectively, treated for recurrent UTI (p=0.073). Twenty (27%) patients in the beta-lactam group required ED re-evaluation or hospital readmission compared to 30 (25%) patients who received a FQ or TMP/SMX (p=0.74). Zero patients had a *Clostridioides difficile* infection.

**Conclusion:**

Oral beta-lactams are as effective as oral FQ and TMP/SMX for the treatment of bacteremia from a urinary source.

**Disclosures:**

**All Authors**: No reported disclosures

